# Bedside Ultrasonographic Measurement of Optic Nerve Sheath Diameter for Assessing Increased Intracranial Pressure: An Observational Study

**DOI:** 10.7759/cureus.86163

**Published:** 2025-06-16

**Authors:** Saurav Shekhar, Raj B Singh, Preeti Sharma, Swapna Lata, Nitin Kumar, Ranjeet Rana De, Amit Kumar

**Affiliations:** 1 Anesthesiology (Trauma and Emergency), Indira Gandhi Institute of Medical Sciences, Patna, IND; 2 Otolaryngology, Patna Medical College & Hospital, Patna, IND; 3 Anesthesia and Critical Care, Indira Gandhi Institute of Medical Sciences, Patna, IND; 4 Emergency Medicine, Indira Gandhi Institute of Medical Sciences, Patna, IND; 5 Radiology, Indira Gandhi Institute of Medical Sciences, Patna, IND

**Keywords:** glasgow coma scale, intracranial pressure, neurological outcome, optic nerve sheath diameter, traumatic brain injury, ultrasonography

## Abstract

Background

Traumatic brain injury (TBI) is a leading cause of disability and mortality, particularly in resource-limited settings where advanced neuromonitoring tools are often unavailable. Elevated intracranial pressure (ICP) is a serious and potentially life-threatening complication of TBI. Traditional methods for monitoring ICP are invasive, expensive, and require specialized expertise. Ultrasonographic measurement of the optic nerve sheath diameter (ONSD) offers a noninvasive, real-time, and radiation-free alternative for assessing ICP.

Objective

This study aimed to evaluate the diagnostic accuracy of bedside ONSD measurement in detecting elevated ICP among neurotrauma patients, using CT imaging as the reference standard. Additionally, it explored the relationship between ONSD measurements, TBI severity, and neurological outcomes.

Methods

A prospective observational study was conducted on 100 adult patients with TBI, in whom bilateral ONSD measurements were obtained using a high-frequency linear ultrasound probe. Based on CT scan findings, patients were classified as having either raised or normal ICP. Statistical analyses included sensitivity, specificity, positive and negative predictive values, receiver operating characteristic curve analysis, and logistic regression to assess the diagnostic and prognostic utility of ONSD.

Results

Raised ICP was observed in 67% of patients, most of whom had moderate to severe TBI. ONSD values increased with injury severity: mild (4.53 ± 0.14 mm), moderate (5.24 ± 0.15 mm), and severe (5.90 ± 0.25 mm). A cutoff value of 5.2 mm demonstrated 95.5% sensitivity, 93.9% specificity, and an area under the curve of 0.96. An ONSD greater than 5.5 mm was strongly associated with poor neurological outcomes (Glasgow Outcome Scale scores 1-3), with an OR of 4.6 (p < 0.001).

Conclusions

Bedside ultrasonographic measurement of ONSD is a reliable and accurate method for detecting elevated ICP in TBI patients. It correlates strongly with both injury severity and clinical outcomes, making it a valuable tool in emergency and intensive care settings. Further studies comparing this technique with invasive monitoring methods and evaluating long-term outcomes are warranted.

## Introduction

Traumatic brain injury (TBI) significantly contributes to mortality and long-term disability, placing a considerable burden on healthcare systems, particularly in developing countries. In India alone, approximately 1.5 to 2 million people sustain a TBI each year [1]. These patients present to the ED with varying degrees of severity, depending on the nature of the injury and the presence of elevated intracranial pressure (ICP). Raised ICP is defined as a sustained pressure exceeding 20 mm Hg [2]. Rapid and accurate evaluation of ICP is critical in these patients to enable timely interventions and maintain adequate cerebral perfusion, thereby potentially reducing morbidity and mortality by preventing secondary brain injury.

Neurological monitoring is essential in evaluating brain function and integrity, involving parameters such as ICP, cerebral perfusion pressure, cerebral blood flow, brain metabolism, oxygen utilization, thermoregulation, and electrical brain activity [3]. While direct methods of ICP measurement, such as epidural bolts, intraventricular catheterization, microdialysis catheters, intraparenchymal transducers, and lumbar puncture, remain the gold standard, they are invasive and carry risks, including catheter-related infections and hemorrhage. These procedures are also contraindicated in patients with coagulopathies and require a high level of technical expertise [4,5].

Point-of-care ultrasound measurement of the optic nerve sheath diameter (ONSD) has emerged as a cost-effective, noninvasive, and radiation-free bedside method for evaluating ICP and detecting intracranial hypertension. A key advantage of this technique is its ability to provide real-time, reproducible data. It is especially valuable in hemodynamically unstable patients in ICUs, as it eliminates the risks associated with transferring patients to a CT scan facility [5,6].

The optic nerve, which extends from the retina to the optic chiasm, is surrounded by cerebrospinal fluid within the subarachnoid space. Because the optic nerve sheath is contiguous with the intracranial subarachnoid space, increases in ICP result in corresponding pressure changes in the intraorbital space. This leads to distension of the optic nerve sheath, particularly in the retrobulbar segment - a hallmark of elevated ICP in TBI. Such distension manifests as an increase in ONSD, making it a potential noninvasive indicator of raised ICP [7-9].

The present study was conducted to evaluate the diagnostic accuracy of bedside ultrasound-guided ONSD measurement for detecting elevated ICP and to compare these results with CT-based findings in neurocritical patients. The primary aim was to assess the clinical utility and accuracy of ONSD as a surrogate marker for changes in ICP.

The primary outcome of this study was to determine the diagnostic accuracy of ONSD in identifying raised ICP, using CT imaging as the reference standard. Diagnostic performance was assessed through calculations of sensitivity, specificity, positive predictive value (PPV), and negative predictive value (NPV). A receiver operating characteristic (ROC) curve was plotted, and the area under the curve (AUC) was calculated to evaluate overall accuracy. An optimal cutoff value for ONSD was determined using Youden’s Index. The secondary outcome was to explore the correlation between ONSD and the clinical severity of ICP, as well as the predictive value of ONSD for mortality and neurological outcomes in patients with TBI.

## Materials and methods

Following institutional ethical approval and registration with the Clinical Trials Registry of India (CTRI/2024/12/077523), this prospective observational study enrolled 100 adult patients with TBI after obtaining written informed consent. The study was conducted in the trauma and emergency ICU of a tertiary care medical college in India between December 2024 and April 2025.

Inclusion and exclusion criteria

Patients aged 18-70 years of either gender presenting with TBI, including intracerebral hemorrhage, stroke, and diffuse axonal injury (DAI), were included. Patients were excluded if they had a prior history of ocular trauma, structural ophthalmologic or optic nerve pathology, or eye or orbital diseases (e.g., glaucoma, lens opacity, or penetrating injury), were younger than 18 or older than 70 years, or if their legal guardians declined participation.

Clinical data collection

Demographic data (age and gender), vital signs (heart rate, respiratory rate, and blood pressure), and clinical status were recorded at presentation. The severity of brain injury was assessed using the Glasgow Coma Scale (GCS), and initial non-contrast CT scans were performed to evaluate intracranial pathology. Neurological outcomes were evaluated at discharge using the Glasgow Outcome Scale (GOS). The collected data were analyzed to examine the association between ONSD and other clinical parameters.

ONSD measurement

Bilateral ONSD measurements were performed by emergency physicians trained in point-of-care ultrasound. All operators underwent standardized training to reduce interoperator variability, and a uniform scanning protocol was followed. In a subset of patients, a second blinded operator repeated measurements to assess reliability. A high-frequency linear ultrasound probe was used with the patient in a supine position, head elevated to 35°, and eyes gently closed. The probe was placed over the upper eyelid using minimal pressure until the optic nerve was visualized as a hypoechoic tubular structure behind the globe. ONSD was measured 3 mm posterior to the retina by drawing a transverse line from the inner edge to the inner edge of the hypoechoic sheath, representing the dura mater. This anatomical point is considered the most responsive to elevated ICP.

CT evaluation of ICP

All patients underwent non-contrast CT imaging of the brain as part of routine clinical evaluation for suspected raised ICP. Findings indicative of elevated ICP included midline shift ≥5 mm, cerebral edema or diffuse brain swelling (loss of gray-white matter differentiation), ventricular dilatation, and presence of intracranial hemorrhage (ICH), mass effect, or herniation. CT scans without these features were categorized as normal ICP. Based on these criteria, patients were classified into two groups: raised ICP or normal ICP.

Statistical analysis

All statistical analyses were performed using GraphPad (GraphPad Software Inc., San Diego, California, USA). The diagnostic accuracy of ONSD was evaluated using sensitivity, specificity, PPV, and NPV. ROC curve analysis was conducted to determine the optimal ONSD cutoff for predicting raised ICP. Descriptive statistics were presented as mean ± SD for continuous variables and as counts with percentages for categorical variables. Group comparisons were made using unpaired Student’s t-test, chi-square test, or Fisher’s exact test, as appropriate. A p-value of ≤0.05 was considered statistically significant.

## Results

A total of 100 patients with TBI were included in our study. The mean age was 40.8 ± 13.5 years, with a predominance of male patients. This distribution aligns with global epidemiological data, often attributed to increased exposure to trauma due to occupational and behavioral factors. The cohort included cases of ICH, ischemic stroke, subarachnoid hemorrhage (SAH), DAI, and mixed pathology. Patients were categorized based on GCS scores into mild, moderate, and severe TBI groups. Among patients with ICH, CT scans revealed raised ICP in 90.9% (20 cases) of those with severe TBI, compared to 0% in the mild group (Fisher’s exact test, p < 0.001). GOS scores were significantly worse in the severe ICH group, with 16 patients (72.7%) showing poor recovery versus 0% in the mild group (chi-square test, p < 0.01).

In patients with ischemic stroke, raised ICP was observed in nine severe cases (81.8%) compared to one mild case (16.7%) (Fisher’s exact test, p = 0.021). Poor GOS outcomes were more common among severe cases (seven patients, 63.6%) than in mild cases (chi-square test, p = 0.038). Among SAH patients, raised ICP was present in four severe cases (80%) (Fisher’s exact test, p = 0.045). GOS outcomes tended to indicate poorer recovery in severe SAH cases, although the small sample size limited statistical power (chi-square test, p = 0.05). In DAI cases, 87.5% (seven patients) of those with severe TBI had poor GOS outcomes, while the remaining showed moderate outcomes (chi-square test, p = 0.013) (Table 1).

**Table 1:** Mean ONSD across different clinical conditions with corresponding GCS classifications and GOS outcome analysis ^* ^Statistically significant p-value (p < 0.05) is denoted by an asterisk. DAI, diffuse axonal injury; GCS, Glasgow Coma Scale; ICH, intracranial hemorrhage; ICP, intracranial pressure; ONSD, optic nerve sheath diameter

Condition	GCS category (n = number of patients in subset)	ONSD measurement (mean ± SD)	Number of patients with CT findings of raised ICP	Neurological outcome based on GOS score			Fisher's exact test (raised ICP)	Chi-square test (GOS outcome)
				Good recovery	Moderate recovery	Poor recovery		
ICH (n = 44)	Mild (8)	4.4 ± 0.2	0	6	2	0	<0.01^*^	<0.01^*^
	Moderate (14)	5.3 ± 0.26	10	3	7	4	<0.01^*^	<0.01^*^
	Severe (22)	5.9 ± 0.41	20	0	6	16	<0.01^*^	<0.01^*^
Ischemic stroke (n = 24)	Mild (6)	4.7 ± 0.1	1	4	2	0	<0.02^*^	< 0.03^*^
	Moderate (7)	5.3 ± 0.2	5	1	3	3	<0.02^*^	<0.03^*^
	Severe (11)	5.8 ± 0.3	9	0	4	7	<0.02^*^	<0.03^*^
Subarachnoid hemorrhage (n = 11)	Mild (2)	4.5	0	2	0	0	<0.04*	<0.05
	Moderate (4)	5.1 ± 0.1	2	2	2	0	<0.04^*^	<0.05
	Severe (5)	5.72 ± 0.2	4	0	1	4	<0.04^*^	<0.05
DAI (n = 13)	Moderate (5)	5.2 ± 0.2	2	0	3	2	<0.01^*^	< 0.01^*^
	Severe (8)	5.9 ± 0.28	8	0	1	7	<0.01^*^	<0.01^*^
Mixed (n = 8)	Moderate (3)	5.2 ± 0.1	1	0	1	2	<0.01^*^	<0.01^*^
	Severe (5)	6.1 ± 0.2	5	0	0	5	<0.01^*^	<0.01^*^

**Figure 1:**
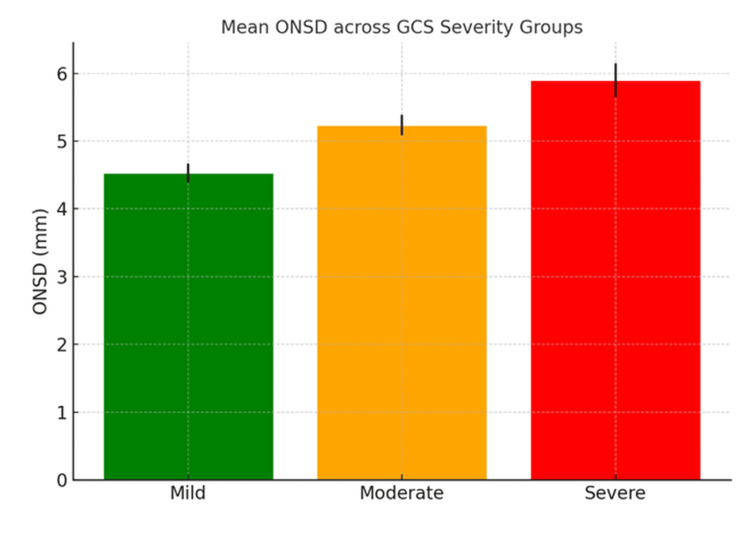
Mean ONSD across GCS severity groups GCS, Glasgow Coma Scale; ONSD, optic nerve sheath diameter

**Figure 2:**
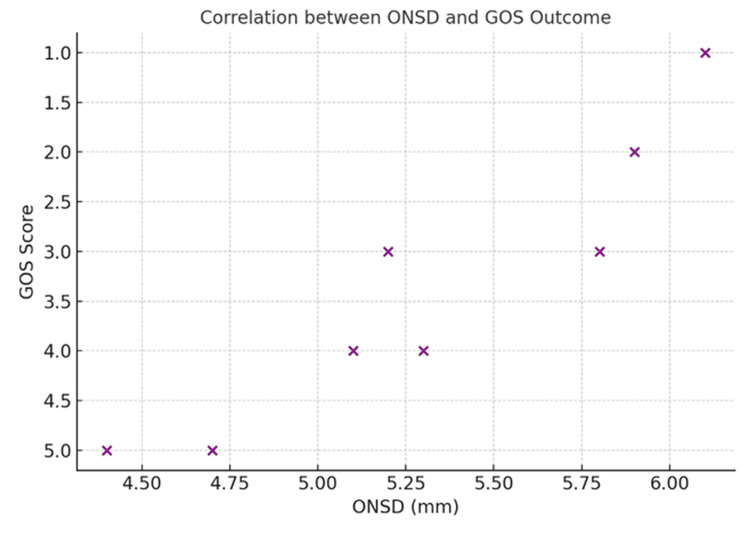
Correlation between ONSD and GOS outcomes GOS, Glasgow Outcome Scale; ONSD, optic nerve sheath diameter

Mean ONSD increased progressively across the GCS severity categories (Figure 1). Raised ICP, as seen on CT scans, was more frequently observed in patients with moderate to severe GCS scores. Neurological outcomes, assessed using the GOS, worsened with both increasing ONSD values and TBI severity (Figure 2). Raised ICP was defined radiologically based on CT findings, including midline shift ≥5 mm, cerebral edema, herniation, and related features. Among the 100 patients included, 67 exhibited CT findings indicative of raised ICP, which was more frequently associated with elevated ONSD values (mean >5.3 mm).

**Figure 3:**
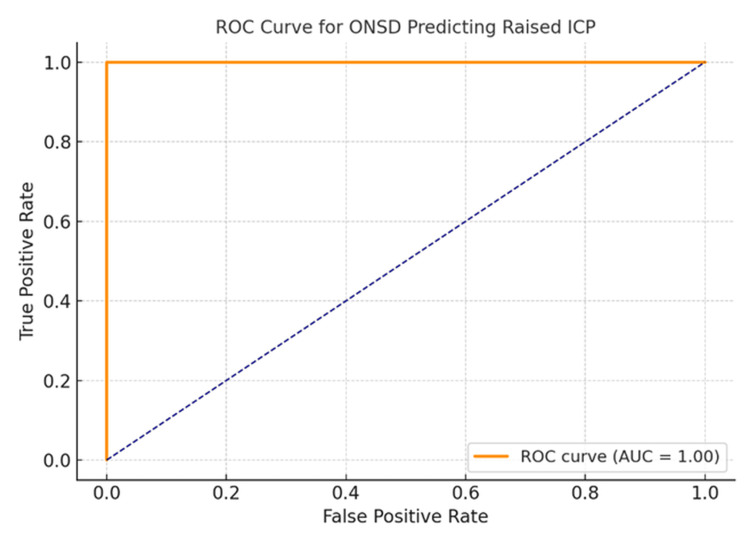
ROC curve for ONSD predicting raised ICP AUC, area under the curve; ICP, intracranial pressure; ONSD, optic nerve sheath diameter; ROC, receiver operating characteristic

**Figure 4:**
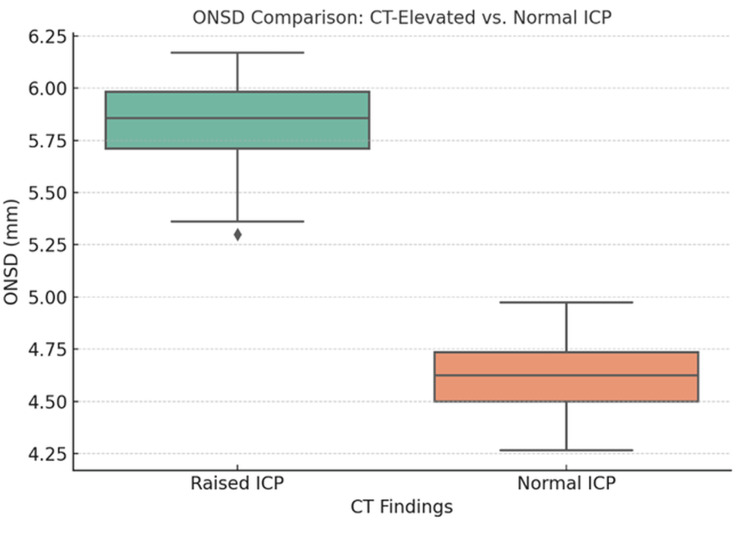
ONSD comparison: CT-elevated vs. normal ICP ICP, intracranial pressure; ONSD, optic nerve sheath diameter

Using a cutoff value of >5.3 mm for ONSD, determined by visual inspection and correlation with clinical outcomes, the following diagnostic parameters were observed: sensitivity 95.5%, specificity 93.9%, PPV 97.0%, and NPV 91.2%. ROC curve analysis demonstrated excellent diagnostic performance, with an AUC of 0.96 (95% CI: 0.92-0.99) (Figure 3). The optimal cutoff for ONSD based on Youden’s Index was 5.2 mm. ONSD measurements positively correlated with TBI severity according to GCS classification: mild: 4.53 ± 0.14 mm; moderate: 5.24 ± 0.15 mm; and severe: 5.90 ± 0.25 mm. ANOVA showed a statistically significant difference in ONSD across GCS levels (p < 0.001), and post hoc Tukey analysis confirmed significant differences between all groups (Figure 4).

ONSD and neurological outcome (GOS)

Patients with ONSD >5.5 mm demonstrated a significantly higher incidence of poor neurological outcomes (GOS 1-3). In particular, all severe TBI cases with ONSD >5.9 mm showed poor recovery. Chi-square analysis confirmed a significant association between ONSD >5.5 mm and poor GOS outcomes (p < 0.001). Logistic regression identified ONSD as an independent predictor of poor neurological outcome, with an OR of 4.6 (95% CI: 2.1-10.3). Severe DAI and mixed pathology cases showed the highest mean ONSD values (5.9-6.1 mm) and the worst outcomes. Conversely, patients with mild injury (ONSD <4.7 mm) showed no signs of raised ICP on CT and experienced good recovery in over 80% of cases.

## Discussion

The present study reaffirms the clinical utility of ONSD as a noninvasive bedside marker for detecting elevated ICP in patients with TBI. Our findings align with existing literature supporting the reliability and diagnostic accuracy of ultrasonographic ONSD measurements in critical care settings.

Kaur et al. [10] reported a mean ONSD of 5.6 ± 0.3 mm in patients with suspected raised ICP, identifying elevated ONSD in 46% of cases, particularly among those with low GCS scores (3-6). Their study demonstrated a diagnostic sensitivity of 93.2% and specificity of 91.1% when ONSD was compared with CT findings, closely mirroring our results. The high PPV (89.1%) and NPV (94.4%) further support the integration of ONSD into initial trauma evaluations.

In a study involving 56 patients undergoing invasive ICP monitoring, Yic et al. [11] found a significant association between ONSD and elevated ICP, with a proposed cutoff of 5.7 mm yielding a sensitivity of 92.9%. Their logistic regression analysis predicted raised ICP with strong statistical significance (p = 0.00803), further validating the role of ONSD as a dependable surrogate marker.

A meta-analysis by Koziarz et al. [12], encompassing over 70 studies and 4,500 patients, reported pooled sensitivity and specificity values of 97% and 86%, respectively, for ONSD in diagnosing raised ICP. Notably, the study highlighted the consistency of ONSD measurements across diverse clinical scenarios, irrespective of operator expertise or patient demographics. These findings emphasize the generalizability and robustness of ONSD in both emergency and ICU settings.

Similarly, Munakomi and Chaulagain [13] observed a strong correlation between increasing ONSD values and both CT progression and GCS deterioration in a cohort of 54 ICU patients. With an AUC of 0.882 for ONSD changes predicting elevated ICP and a specificity approaching 90%, their results closely reflect our study’s ROC analysis and further affirm the predictive value of ONSD in clinical deterioration.

Beyond trauma, Yang et al. [14] evaluated ONSD in septic patients and found significantly larger diameters in those with sepsis-associated encephalopathy. At a cutoff of ≥5.5 mm, ONSD had a sensitivity of 80.4% and specificity of 83.5%. They also reported a strong inverse correlation between ONSD and GCS scores (rs = -0.666), echoing our findings of higher ONSD values in patients with more severe neurological impairment.

Dynamic evidence from Chen et al. [15] further supports ONSD’s clinical relevance. Their study demonstrated significant reductions in ONSD following lumbar puncture, with correlation coefficients ranging from 0.451 to 0.482. These findings indicate that ONSD not only reflects static ICP but can also monitor real-time changes, reinforcing its value in guiding treatment decisions.

Additionally, Shirodkar et al. [16] validated the agreement between ultrasonographic and MRI-based ONSD measurements, reporting an acceptable correlation. This supports the use of sonographic ONSD assessment as a reliable, noninvasive alternative, particularly valuable when advanced imaging modalities are unavailable.

Taken together, these findings underscore that ONSD is a sensitive, specific, and practical tool for identifying raised ICP. Its noninvasive nature, reproducibility, and suitability for bedside application make it especially valuable in resource-limited and emergency care settings where immediate neuroimaging may not be feasible. However, ONSD measurement is operator-dependent and susceptible to technique-related variability, highlighting the need for proper training and standardization.

Limitations

Larger multicenter trials in diverse clinical settings are required to refine optimal ONSD cutoffs and improve the generalizability of findings. Interobserver variability remains a potential limitation, as does the exclusive focus on patients presenting to the ED or ICU, which may limit applicability to outpatient populations. Additionally, the timing of CT imaging may not always reflect dynamic changes in ICP. While CT was used as the reference standard in this study, invasive ICP monitoring remains the gold standard for definitive diagnosis.

## Conclusions

This study demonstrates that bedside ultrasonographic measurement of ONSD is a highly sensitive, specific, and noninvasive method for detecting raised ICP in TBI patients. ONSD values showed a strong correlation with clinical severity, as measured by the GCS, and with radiological evidence of raised ICP on CT. An ONSD cutoff of 5.2 mm provided optimal diagnostic accuracy, while values exceeding 5.5 mm were significantly associated with poor neurological outcomes.

Given its safety, reproducibility, real-time applicability, and ease of use, ONSD ultrasonography holds significant promise as a routine bedside assessment tool in emergency and critical care settings, especially in environments where advanced imaging may not be immediately accessible. Incorporating ONSD into neuromonitoring protocols could enhance early detection of elevated ICP, support timely intervention, and improve overall patient outcomes.
